# Supplementary material for: Deep Sequencing of Small RNA Reveals the Molecular Regulatory Network of AtENO2 Regulating Seed Germination
Source: Int J Mol Sci. 2021 May 11;22(10):5088. doi: 10.3390/ijms22105088 (PMC8151434; doi:10.3390/ijms22105088)
Supplement: Supplementary file 1 [file ijms-22-05088-s001.zip › Supplementary Figures.pdf]

Supplementary Figures

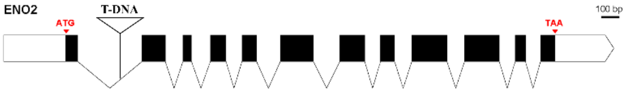

Figure S1. The structure of *ENO2* mutant (*eno2*⁻).

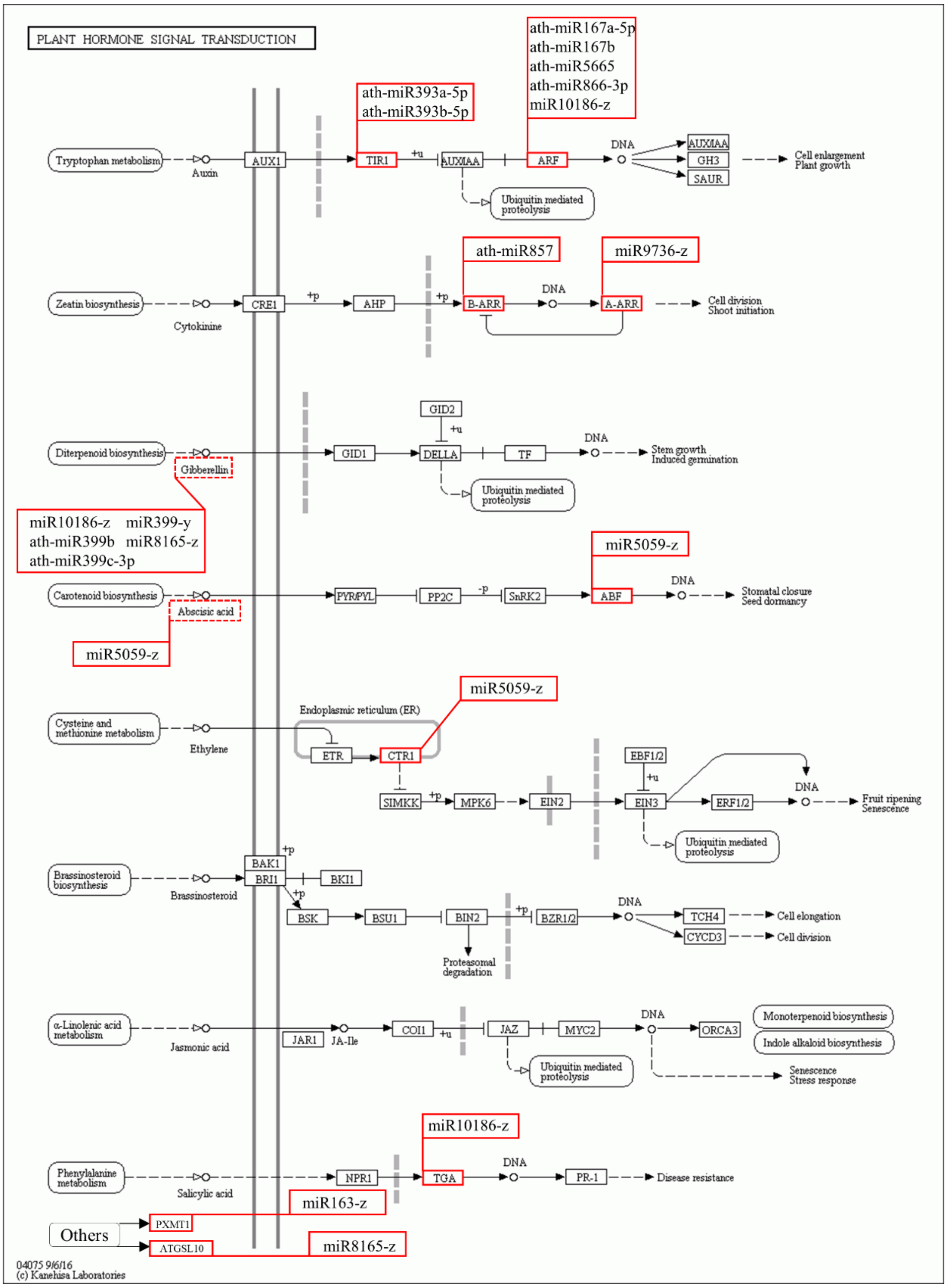

Figure S2. Phytohormone signal transduction pathway and related miRNAs that regulate seed germination.

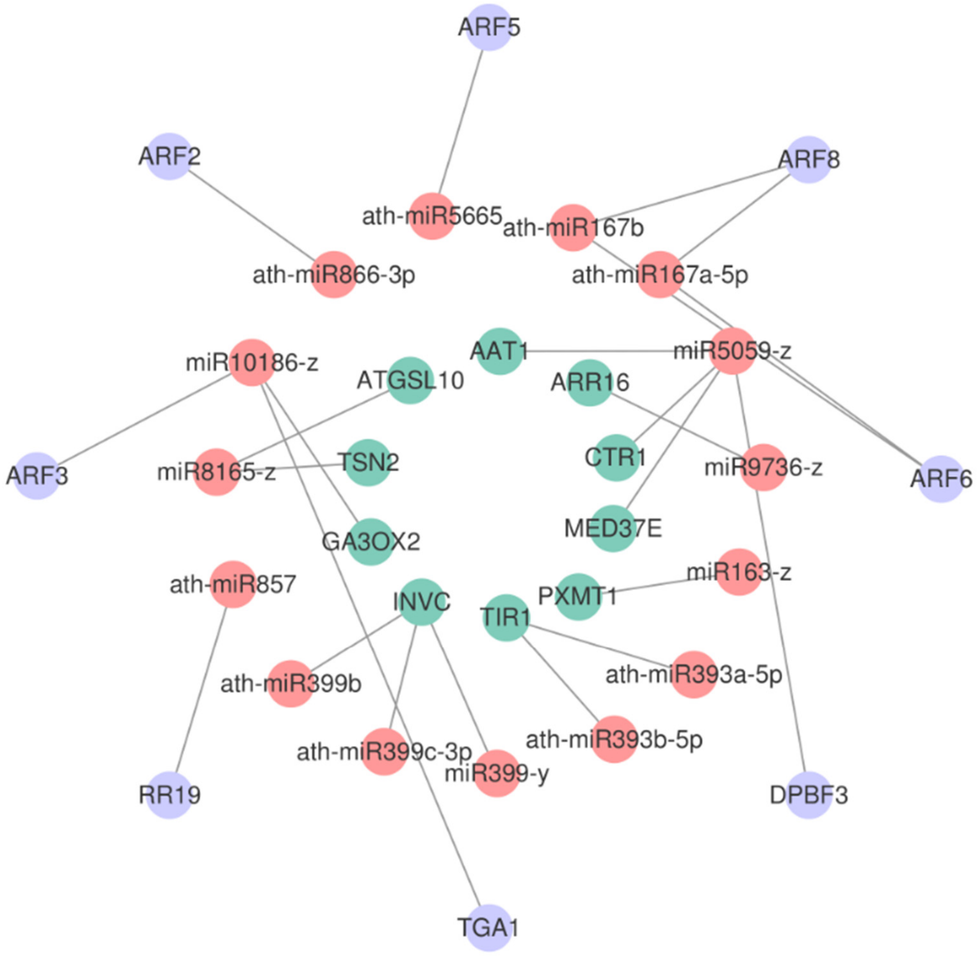

Figure S3. TF-miRNA-gene regulatory network.

Cytoscape (Cytoscape_3.7.1) was used to analyze these miRNAs and their target genes. The red circles represent miRNAs, and the green and purple circles were their target genes. The gene with purple circles were also transcription factors.
